# Effects of Functionalization of TiO_2_ Nanotube Array Sensors with Pd Nanoparticles on Their Selectivity

**DOI:** 10.3390/s140915849

**Published:** 2014-08-27

**Authors:** Sunghoon Park, Soohyun Kim, Suyoung Park, Wan In Lee, Chongmu Lee

**Affiliations:** 1 Department of Materials Science and Engineering, Inha University, 253 Yonghyun-Dong, Incheon 402-751, Korea; E-Mails: aju-ya@hanmail.net (Sun.P.); feel2779@naver.com (S.K.); dswimming@naver.com (Suy.P.); 2 Department of Chemistry, Inha University, 253 Yonghyun-Dong, Incheon 402-751, Korea; E-Mail: wanin@inha.ac.kr

**Keywords:** TiO_2_, nanotube, gas sensor, metal catalyst, acetone

## Abstract

This study compared the responses of Pd-functionalized and pristine titanate (TiO_2_) nanotube arrays to ethanol with those to acetone to determine the effects of functionalization of TiO_2_ nanotubes with Pd nanoparticles on the sensitivity and selectivity. The responses of pristine and Pd-functionalized TiO_2_ nanotube arrays to ethanol gas at 200 °C were ∼2877% and ∼21,253%, respectively. On the other hand, the responses of pristine and Pd-functionalized TiO_2_ nanotube arrays to acetone gas at 250 °C were ∼1636% and 8746% respectively. In the case of ethanol sensing, the response and recovery times of Pd-functionalized TiO_2_ nanotubes (10.2 and 7.1 s) were obviously shorter than those of pristine TiO_2_ nanotubes (14.3 and 8.8 s), respectively. In contrast, in the case of acetone sensing the response and recovery times of Pd-functionalized TiO_2_ nanotubes (42.5 and 19.7 s) were almost the same as those of pristine TiO_2_ nanotubes (47.2 and 17.9 s). TiO_2_ nanotube arrays showed the strongest response to ethanol and Pd functionalization was the most effective in improving the response of TiO_2_ nanotubes to ethanol among six different types of gases: ethanol, acetone, CO, H_2_, NH_3_ and NO_2_. The origin of the superior sensing properties of Pd-functionalized TiO_2_ nanotubes toward ethanol to acetone is also discussed.

## 1. Introduction

Titanate (TiO_2_) is one of the most widely studied semiconductor materials. Formation of TiO_2_ nanotubes was reported for the first time by Hoyer [1]. Anodic formation of titania nanotubes was demonstrated for the first time by Zwilling *et al.* [2,3]. Over the past two decades, a range of applications including dye-sensitized solar cells, batteries, photocatalysts and chemical sensors have been explored [4–7]. TiO_2_ nanotube (NT) arrays have been used to detect O_2_, NO_2_, H_2_, CO, ammonia, formaldehyde, acetone, ethanol, chloroform, humidity and other gases [6,8–20]. TiO_2_ NT gas sensors showed high sensitivity and a rapid response at relatively low temperatures [7,20].

The functionalization of semiconductor gas sensors with metal catalysts is one of the most commonly used techniques for enhancing the sensitivity of gas sensors. The electrical response of a gas sensor to the target gas depends strongly on the efficiency of catalytic reactions of the surface of gas sensor material with the target gas. The sensitivity of gas sensors can be enhanced considerably by modifying the catalytic activity of the gas sensor material using metal catalysts such as Pd, Pt, Au and Ag. Table 1 lists some references for metal catalyst-functionalized TiO_2_ nanotube sensors in the literature [21–27]. Four kinds of metal catalyst-functionalized TiO_2_ nanotube arrays were assessed to detect a range of gases, but their sensing properties toward ethanol gas were not reported. The mechanism through which the sensitivity of a gas sensor is enhanced by functionalizing the sensor material with metal catalysts is well established [28–31]. Basically, a combination of electronic and chemical mechanisms is used to explain the enhanced sensitivity. Nevertheless, the effects of metal catalysts on the selectivity of sensors are not completely understood.

In this study, the sensing properties of pristine and Pd-functionalized TiO_2_ nanotube array sensors toward ethanol and acetone were compared to determine the effects of the metal catalyst on the sensitivity and selectivity of the sensors. The differences in the sensitivity and sensing speed of pristine and Pd-functionalized TiO_2_ nanotube arrays between the two gases were analyzed based on the sensing mechanism of metal catalyst-functionalized oxide semiconductor nanostructures. Pd has been most commonly adopted as a metal catalyst because of its excellent catalytic behavior.

## 2. Results and Discussion

**Figure 1.**
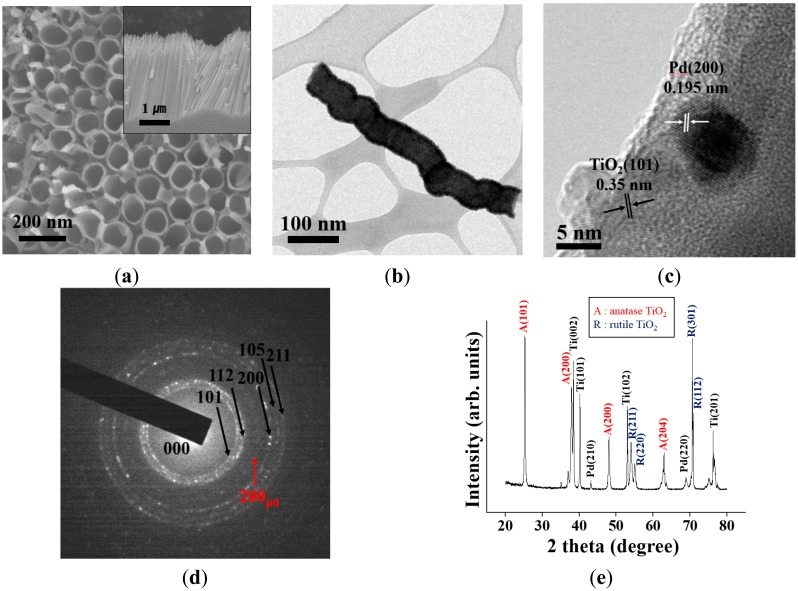
(**a**) Scanning electron microscopy image of Pd-functionalized TiO_2_ nanotubes; (**b**) Low-magnification transmission electron microscopy (TEM) image of a typical Pd-functionalized TiO_2_ nanotube; (**c**) High-resolution TEM image and (**d**) Corresponding selected area electron diffraction pattern of a typical Pd-functionalized TiO_2_ nanotube; (**e**) X-ray diffraction pattern of Pd-functionalized TiO_2_ nanotubes.

**Figure 2.**
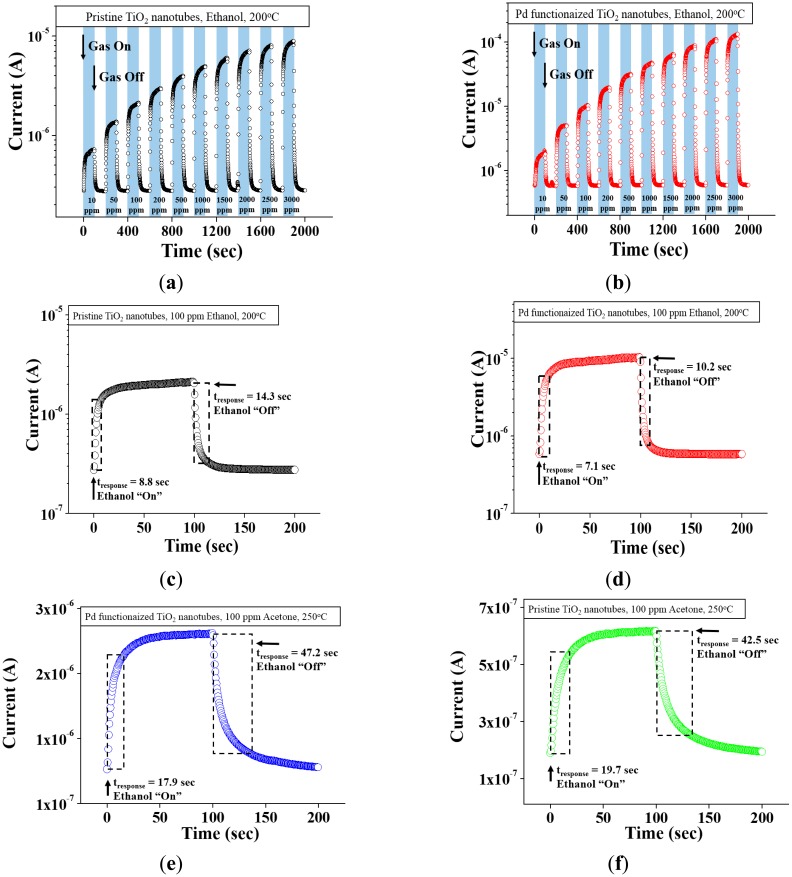
Electrical responses of the gas sensors fabricated from (**a**) pristine TiO_2_ nanotubes and (**b**) Pd-functionalized TiO_2_ nanotubes to 10–3000 ppm C_2_H_5_OH gas at 200 °C; (**c**) Enlarged part of the pristine TiO_2_ nanotube curve in Figure 2a at 100 ppm C_2_H_5_OH; (**d**) Enlarged part of the Pd-functionalized nanotube curve in Figure 2b at 100 ppm C_2_H_5_OH at 200 °C; (**e**) Electrical response of pristine TiO_2_ nanotubes to 100 ppm CH_3_COCH_3_ at 250 °C; (**f**) Electrical response of Pd-functionalized TiO_2_ nanotubes to 100 ppm CH_3_COCH_3_ at 250 °C. Note: The sensing test temperature for C_2_H_5_OH differs from that for CH_3_COCH_3_.

Figure 1a and the inset in Figure 1a show the top view and cross-sectional view scanning electron microscopy (SEM) images, respectively, of a Pd-functionalized TiO_2_ nanotube array synthesized by anodization followed by annealing at 500 °C for 2 h. The length and diameter of the titanium nanotube were 300–400 nm and 70–120 nm, respectively. Figure 2 shows X-ray diffraction (XRD) patterns of the sample (annealed at 500 °C) obtained at an anodizing voltage of 50 V. This suggests that the phases present are anatase TiO_2_ (JCPDS Card 89-4921), titanium (Ti) (JCPDS Card 89-5009) and palladium (Pd) (JCPDS Card 89-4897). The as-synthesized TiO_2_ nanotube array sample was amorphous, but crystallized after annealing at 500 °C and coating with Pd. According to the literature, TiO_2_ crystallizes above 300 °C [32,33]. The clear sharp peaks observed in the XRD patterns indicate the presence of a crystalline phase. According to the literature, with increasing calcination temperature, TiO_2_ crystallizes into two phases: rutile and anatase with anatase dominant at lower temperatures. After annealing at 500 °C for 2 h, TiO_2_ nanotubes were crystallized completely, but stable rutile and metastable anatase TiO_2_ phases coexisted. In addition to the peaks from TiO_2_ four peaks characteristic of metal Ti and two small reflection peaks characteristic of metal Pd were identified, which were indexed as the (002), (101), (102) and (201) reflections from tetragonal-structured Ti and the (200) and (220) reflections from face-centered cubic-structured Pd. The presence of Pd nanoparticles on the TiO_2_ nanotube surface was confirmed further by transmission electron microscopy (TEM). High-resolution TEM (HRTEM) image (Figure 1d) revealed a spherical Pd particle with a diameter of ∼7 ± 4 nm on the surface of TiO_2_ nanotube. A close examination revealed two different fringe patterns: one with a spacing of 0.195 nm corresponding to the interplanar spacing of the Pd (200) lattice plane and the other with a spacing of 0.35 nm corresponding to the interplanar spacing of the TiO_2_ (101) lattice plane. The corresponding selected area electron diffraction (SAED) pattern (Figure 1e) showed a concentric ring pattern, suggesting that both TiO_2_ nanotubes and Pd nanoparticles were polycrystalline, even if they showed local fringe patterns. The concentration of Pd in the Pd-functionalized TiO_2_ nanotube array was estimated to approximately 1% based on the low-magnification TEM and HRTEM images.

Figure 2a,b shows the dynamic electric responses of pristine and Pd-functionalized TiO_2_ nanotube arrays, respectively, to a reducing gas ethanol pulses at concentrations of 10, 50, 100, 200, 500, 1000, 1500, 2000, 2500 and 3000 ppm at 200 °C. The current increases reversibly upon each ethanol pulse. The electrical behavior of the sensors was consistent, recovering their original currents without hysteresis after repeated exposure to different ethanol gas concentrations.

The pristine TiO_2_ nanotube arrays showed responses of approximately 235, 468, 691, 986, 1334, 1657, 2009, 2355, 2693 and 2877% to 10, 50, 100, 200, 500, 1000, 1500, 2000, 2500 and 3000 ppm ethanol, respectively. In contrast, the Pd-functionalized TiO_2_ nanotube arrays showed responses of approximately 297, 796, 1590, 3132, 4997, 7196, 9984, 14,079, 17,072 and 21,253% to 10, 50, 100, 200, 500, 1000, 1500, 2000, 2500 and 3000 ppm ethanol, respectively. Pd-functionalization increased the response to 3000 ppm ethanol gas by more than seven fold.

Figure 2c,d shows the enlarged parts of the dynamic response curves of pristine and Pd-functionalized TiO_2_ nanotube arrays in Figure 2a,b, respectively, to 100 ppm ethanol at 200 °C drawn to illustrate the moments of gas input and gas stop. Similarly, Figure 2e,f presents the response curves of pristine and Pd-functionalized TiO_2_ nanotube arrays, respectively, at 100 ppm acetone at 250 °C. The sensing data at 200 °C and 250 °C for ethanol and acetone, respectively, are shown in these figures because the best sensing data was obtained at those temperatures. The responses of pristine and Pd-functionalized TiO_2_ nanotube arrays to ethanol gas at 200 °C were ∼986% and ∼3132%, respectively (Supporting Information, Table S1). On the other hand, the responses of pristine and Pd-functionalized TiO_2_ nanotube arrays to acetone gas at 250 °C were ∼473% and 1152%, respectively. In the case of ethanol sensing, the response time and recovery time of Pd-functionalized TiO_2_ nanotubes (10.2 and 7.1 s) were shorter than those of pristine TiO_2_ nanotubes (14.3 and 8.8 s), respectively. In contrast, the sum of the response time and recovery time of Pd-functionalized TiO_2_ nanotubes (65.1 s) to acetone was similar to that of pristine TiO_2_ nanotubes (62.2 s). A comparison of Figure 2c with Figure 2e showed that both the response and recovery times of pristine TiO_2_ nanotubes toward ethanol gas were significantly shorter than toward acetone gas. On the other hand, a comparison of Figure 2d with Figure 2f showed that both the response time and recovery time of Pd-functionalized TiO_2_ nanotubes toward ethanol gas were also far shorter than toward acetone gas. The following conclusion can be extracted from the above sensing data: Functionalization of TiO_2_ nanotubes with Pd enhances both the sensitivity and sensing speed significantly toward ethanol gas, whereas it enhances only the sensitivity toward acetone gas.

**Figure 3.**
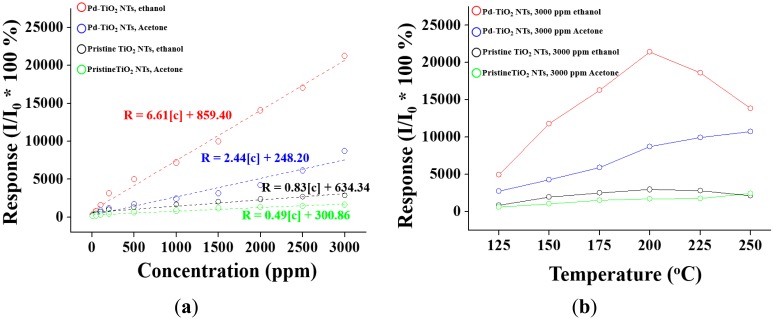
(**a**) Responses of pristine and Pd-functionalized TiO_2_ nanotubes to ethanol or acetone gas as a function of the ethanol or acetone concentration; (**b**) Responses of pristine and Pd-functionalized TiO_2_ nanotubes to ethanol or acetone gas as a function of temperature.

Figure 3a shows the responses calculated from Figure 2a as a function of ethanol concentration. A linear relationship was observed between the response and ethanol concentration in the ethanol gas concentration range below 3000 ppm. The response of a semiconductor is commonly expressed as *R* = A [*C*]*^n^* + B, where A and B, *n* (usually equal to 1), and [*C*] are constants, exponent, and the target gas concentration, respectively [34]. Data fitting resulted in the following equations for responses to ethanol: *R* = 0.83 [*C*] + 634.34 and *R* = 6.61 [*C*] + 859.4 for pristine and Pd-functionalized TiO_2_ nanotubes, respectively. Data fitting also gave the equations for the responses to acetone: *R* = 0.49 [*C*] + 300.86 and *R* = 2.44 [*C*] + 248.20 for pristine and Pd-functionalized TiO_2_ nanotubes, respectively. A comparison of the response curves showed that both the pristine and Pd-functionalized TiO_2_ nanotubes had higher sensitivity to ethanol than to acetone. The Pd-functionalized TiO_2_ nanotubes showed a superior response and a greater increasing rate of the response to ethanol or acetone gas concentration to pristine TiO_2_ nanotubes. The difference in the response of the pristine TiO_2_ nanotubes between ethanol and acetone is not so big, but the difference in response of the Pd-functionalized TiO_2_ nanotubes between ethanol and acetone was much larger.

Figure 3b shows the responses of the pristine and Pd-functionalized TiO_2_ nanotubes to ethanol and acetone as a function of temperature. The pristine and Pd-functionalized TiO_2_ nanotubes showed the strongest responses to ethanol and Pd-functionalized TiO_2_ nanotubes showed a higher response ratio compared to pristine TiO_2_ nanotubes at 200 °C. In addition, Pd-functionalized TiO_2_ nanotubes showed the strongest responses to ethanol at 250 °C. Attempts were made to measure the responses to ethanol and acetone at higher temperatures, but they failed because of the excessive noise in the dynamic response curve at the temperatures.

**Figure 4.**
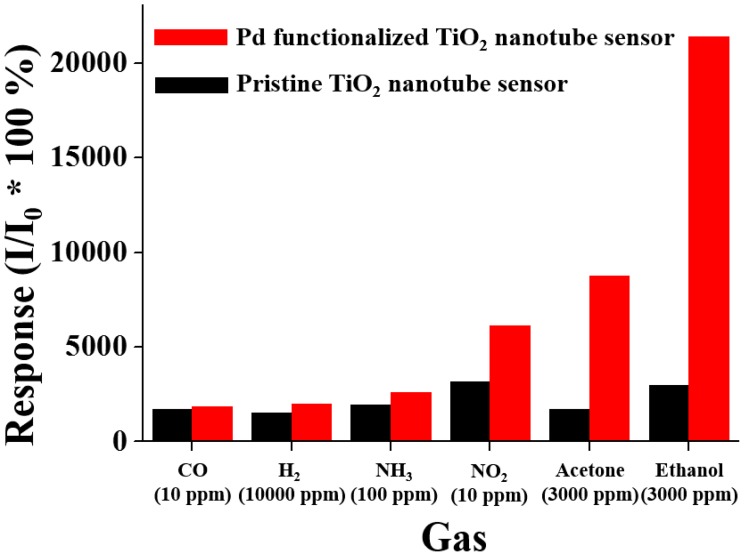
Comparison of responses of pristine and Pd-functionalized TiO_2_ nanotubes to different gases.

Over the past two decades TiO_2_ nanotube arrays were mostly studied to detect hydrogen [18,35,36]. They were commonly operated at temperatures as high as several hundred degrees Celsius, because of their unsatisfactory sensing performances at low temperatures. However, the high operating temperatures are not desirable for many applications, particularly those involving flammable environments or requiring low power operation. Titania nanotube arrays were also used to fabricate humidity sensors [19] and low-temperature oxygen sensors operating at 50–300 °C [20]. On the other hand, studies on the sensing properties of TiO_2_ nanotube arrays to other gases such as ethanol, acetone, CO, NH_3_ and NO_2_ have been relatively fewer compared to H_2_, O_2_ and humidity. We compared the responses of pristine and Pd-functionalized TiO_2_ nanotubes to ethanol with those to other gases as shown in Figure 4 to see the selectivity of TiO_2_ nanotubes in gas sensing. The responses of the pristine and Pd-functionalized TiO_2_ nanotubes to ethanol were far higher than those to other gases, respectively. The responses of the pristine and Pd-functionalized TiO_2_ nanotubes to acetone were far lower than those to ethanol, even if they were higher than the responses to other gases such as H_2_, CO, NH_3_ and NO_2_. At present, it is not well understood why pristine and Pd-functionalized TiO_2_ nanotubes showed stronger responses to ethanol than to hydrogen. We surmise that it is due to relatively low sensing test temperature (200 °C). If the sensing test had been done at a far higher temperature, it would have given different results. In particular, the ratio of the response of Pd-functionalized TiO_2_ nanotubes to that of pristine TiO_2_ nanotubes is the highest for ethanol (∼8.8), the next highest for acetone (∼2.8) and less than 2.0 for other four gases (Supporting Information, Table S2), suggesting that Pd functionalization is most effective in improving the response of TiO_2_ nanotubes to ethanol among the six different kinds of gases.

Upon exposure to air the oxygen species (O_2_^−^, O^−^ and O^2−^) are adsorbed by the TiO_2_ surface, which creates a depletion layer in the surface region of TiO_2_ nanotubes by extracting electrons from the conduction band of TiO_2_ as follows [37]:

(1) $${\text{O}}_{2}\left(\text{air}\right)+{2\text{e}}^{-}={2\text{O}}^{-}\left(\text{film surface}\right)$$

Upon exposure to ethanol (C_2_H_5_OH) gas a large amount of electrons are produced through the following reactions [38], returning to TiO_2_ and then transferring to Pd.

(2) $${\text{CH}}_{3}{\text{CH}}_{2}\text{OH}\left(\text{gas}\right)\to {\text{CH}}_{3}{\text{CH}}_{2}\text{OH}\left(\text{ads}\right)$$

(3) $${\text{CH}}_{3}{\text{CH}}_{2}\text{OH}\left(\text{ads}\right)+{6\text{O}}^{-}\left(\text{ads}\right)\to {2\text{CO}}_{2}\left(\text{gas}\right)+{3\text{H}}_{2}\text{O}\left(\text{gas}\right)+{6\text{e}}^{-}$$

These reactions reduce the electron depletion layer width in the TiO_2_ surface region.

A combination of electronic and chemical mechanisms is commonly used to explain the metal catalyst-induced enhancement of sensitivity [39–41]. In the electronic mechanism, the enhanced sensitivity was based on the modulation of the width of the depletion layer formed around each Pd particle or the conduction channel due to changes in the oxidation state of the Pd accompanying oxygen adsorption and desorption [29]. Pd nanoparticles act as electron acceptors on TiO_2_ nanotube surfaces, resulting in a further decrease in depletion layer width. Consequently, the change in current is larger in Pd-functionalized TiO_2_ nanotubes than in pristine TiO_2_ nanotubes, leading to enhanced response. On the other hand, in the chemical mechanism, the enhanced sensitivity is based on the excellent catalytic dissociation ability of Pd. Pd activates the catalytic dissociation of oxygen and ethanol (or acetone) molecules because it is a far superior dissociation catalyst to TiO_2_. Of these two mechanisms the further enhancement in the response to ethanol by Pd-functionalization than to acetone could be explained by the electronic mechanism as follows:

The amount of electrons (6e^−^) produced by the oxidation reaction of 1 mol of ethanol according to Equation (2) is larger than that (4e^−^) produced by the oxidation reaction of the same amount (1 mol) of acetone (CH_3_COCH_3_) according to Equation (5) [42]:

(4) $${\text{CH}}_{3}{\text{COCH}}_{3}\left(\text{gas}\right)\to {\text{CH}}_{3}{\text{COCH}}_{3}\left(\text{ads}\right)$$

(5) $${\text{CH}}_{3}{\text{COCH}}_{3}\left(\text{ads}\right)+2{{\text{O}}_{2}}^{-}\left(\text{ads}\right)\to {\text{C}}^{+}{\text{H}}_{3}+{\text{CO}}_{2}+{\text{CH}}_{3}{\text{O}}^{-}+{4\text{e}}^{-}$$

Therefore, a narrower depletion layer is created upon exposure to ethanol than to acetone, assuming that the amounts and concentrations of the two target gases are the same.

## 3. Experimental Section

Anodization was performed to synthesize TiO_2_ nanotube arrays using a conventional two-electrode cell system. The as-treated Ti plates were used as the working electrode and a platinum sheet (2 × 1.5 cm^2^) with a platinum wire was used as the counter-electrode. The anodization potential (anodization voltage = 60 V, current = 1–3 mA) was applied using a potentiostat interfaced with a computer. The electrolyte (dimethyl sulfoxide (DMSO) + 2 vol % HF (48%)) was stirred with a magnetic flea during the anodization process. Details of the synthesis procedure of TiO_2_ nanotube arrays are reported elsewhere [43]. Subsequently, the synthesized TiO_2_ nanotube array were immersed in ethanol/(10 mM) PdCl_2_ solution and irradiated with UV laser (λ = 254 nm, I = 1.2 mW/cm^2^) for 30 min. To induce crystallization of TiO_2_ nanotube arrays and allow the Pd thin films on the nanotube surfaces to agglomerate into Pd nanoparticles, the products (Pd-capped TiO_2_ nanotubes) were annealed at 500 °C in an O_2_ atmosphere (flow rate = 30 sccm) for 2 h. The collected nanotube array samples were examined by scanning electron microscopy (SEM, Hitachi S-4200, Tokyo, Japan), transmission electron microscopy (TEM, Philips CM-200, Eindhoven, The Netherland) and X-ray diffraction (XRD, Philips X'pert MRD diffractometer, Eindhoven, The Netherland). The crystallographic structure was determined by glancing angle XRD with Cu-Kα radiation (0.15406 nm) at a scan rate of 4°/min, and a 0.5° glancing angle with a rotating detector.

For the sensing measurement, gold wires were connected directly to both the electrodes and a TiO_2_ nanotube array synthesized on a Ti thin foil substrate using Ag paste. The gas sensing properties of a single TiO_2_ nanotube sensor measured using a home-built computer-controlled characterization system consisting of a test chamber, a sensor holder, a Keithley 2602 source meter, mass flow controllers and a data acquisition system. The test gas was mixed with dry air to achieve the desired concentration and the flow rate was maintained at 200 sccm using mass flow controllers. The current flowing through the samples was measured at 200 or 250 °C using a Keithley 2602 source meter under 50% relative humidity (RH). A given amount of C_2_H_5_OH (>99.99%) gas was injected into the testing tube through a microsyringe to obtain a C_2_H_5_OH concentration of 10–3000 ppm, and the electrical current in the nanotubes was monitored. The response of the TiO_2_ nanotube sensors is defined as (*I*_g_ − *I*_a_)/*I*_a_, where *I*_a_ and *I*_g_ are the electrical currents in the sensors in air and target gas, respectively. Details of the arrangement of the electrodes in the TiO_2_ nanotube gas sensor and the sensing test procedure are reported elsewhere [43]. The response time is defined as the time required for the variation in electrical resistance to reach 90% of the equilibrium value after injecting the gas and the recovery time is defined as the time needed for the sensor to return to 90% above the original resistance in air after removing the gas.

## 4. Conclusions

The responses of pristine (∼2877%) and Pd-functionalized (∼21,253%) TiO_2_ nanotube arrays to ethanol gas at 200 °C were higher than those of pristine (∼1636%) and Pd-functionalized (∼8746%) TiO_2_ nanotube arrays, respectively, to acetone gas at 250 °C. In the case of ethanol sensing, the sensing speed of Pd-functionalized TiO_2_ nanotubes were shorter than that of pristine TiO_2_ nanotubes, whereas in the case of acetone sensing, the sensing speed of Pd-functionalized TiO_2_ nanotubes was the same as that of pristine TiO_2_ nanotubes. TiO_2_ nanotube arrays showed the strongest response to ethanol and Pd functionalization was the most effective in improving the response of TiO_2_ nanotubes to ethanol among six different types of gases: ethanol, acetone, CO, H_2_, NH_3_ and NO_2_. Of the electronic and chemical mechanisms, the further enhancement in response to ethanol rather than acetone by Pd-functionalization can be explained only by electronic mechanism. The amount of electrons (6e^−^) produced by the oxidation reaction of 1 mol of ethanol is larger than that (4e^−^) produced by the oxidation reaction of the same amount (1 mol) of acetone (CH_3_COCH_3_), resulting in superior sensing properties toward ethanol to acetone.

## Supplementary Material



## Figures and Tables

**Table 1.** Summary of the metal catalyst-functionalized TiO_2_ nanotube gas sensors in the literature.

**Type of Nanostructures**	**Functionalization Material**	**Gas**	**Reference**
TiO_2_ nanotube arrays	Pt	SO_2_SOF_2_, SO_2_F_2_	[21]
Anodic TiO_2_ nanotube films	Pt, Pd	H_2_	[22]
TiO_2_ nanotubes	Ni, Pd	CO_2_, methane	[23]
TiO_2_ nanofilms	Pd	H_2_	[24]
TiO_2_ nanotube arrays	Au	CO	[25]
TiO_2_ nanotubes arrays	Au	NO_2_	[26]
TiO_2_ nanotubes	Au	toluene	[27]

## References

[1] Hoyer P. (1996). Formation of a Titanium Dioxide Nanotube Array. Langmuir.

[2] Zwilling V., Darque-Ceretti E. Characterization of porous and compact oxide films on titanium and Ti-6A1-4V. Caracterisation d'oxydes anodiques poreux et compacts de titane et de Ta6V. http://rocks.ensmp.fr/cgi-bin/koha/opac-detail.pl?biblionumber=39894.

[3] Zwilling V., Darque-Ceretti E., Boutry-Forveille A., David D., Perrin M.Y., Aucouturier M. (1999). Structure and physicochemistry of anodic oxide films on titanium and Ta6V alloy. Surf. Interface Anal..

[4] Natarajan T.S., Natarajan K., Bajaj H.C., Tayade R.J. (2011). Energy Efficient UV-LED Source and TiO_2_ Nanotube Array-Based Reactor for Photocatalytic Application. Ind. Eng. Chem. Res..

[5] Smith Y., Sarma B., Mohanty S.K., Misra M. (2012). Light-assisted anodized TiO_2_ nanotube arrays. ACS Appl. Mater. Inter..

[6] Li Z.H., Ding D.Y., Liu Q., Ning C.Q. (2013). Hydrogen Sensing with Ni-Doped TiO_2_ Nanotubes. Sensors.

[7] Galstyan V., Comini E., Faglia G., Sberveglieri G. (2013). TiO_2_ Nanotubes: Recent Advances in Synthesis and Gas Sensing Properties. Sensors.

[8] Evi G.S., Hyodo T., Shimizu Y., Egashira M. (2002). Synthesis of Mesoporous TiO_2_-Based Powders and their Gas-sensing Properties. Sens. Actuators B Chem..

[9] Ruiz A.M., Sakai G., Cornet A., Shimanoe K., Morante J.R., Yamazoe N. (2003). Cr-Doped TiO_2_ Gas Sensor for Exhaust NO_2_ Monitoring. Sens. Actuators B Chem..

[10] Seo M.H., Yuasa M., Kida T., Huh J.S., Yamazoe N., Shimanoe K. (2009). Detection of Organic Gases Using TiO_2_ Nanotube-Based Gas Sensors. Procedia Chem..

[11] Lin S., Li D., Wu J., Li X., Akbar S.A. (2011). A Selective Room Temperature Formaldehyde Gas Sensor Using TiO_2_ Nanotube Arrays. Sens. Actuators B Chem..

[12] Yun H. (2005). Preparation of NO_2_ Gas Sensor Based on TiO_2_ Nanotubes. Master's Thesis.

[13] Mor G.K., Varghese O.K., Paulose M., Ong K.G., Shankar K., Grimes C.A. (2006). A Review on Highly Ordered, Vertically Oriented TiO_2_ Nanotube Arrays: Fabrication, Material Properties, and Solar Energy Applications. Sol. Energy. Mater. Sol. Cells.

[14] Nakagawa H., Yamamoto N., Okazaki S. (2003). A Room-temperature Operated Hydrogen Leak Sensor. Sens. Actuators B Chem..

[15] Grimes C.A. (2007). Synthesis and Application of Highly Ordered Arrays of Nanotubes. J. Mater. Chem..

[16] Allam N.K., Grimes C.A. (2008). Effect of Cathode Material on the Morphology and Photoelectrochemical Properties of Vertically Oriented TiO_2_ Nanotube Arrays. Sol. Energy. Mater. Sol. Cells..

[17] Yang L.X., Yang W.Y., Cai Q.Y. (2007). Well-Dispersed PtAu Nanoparticles Loaded into Anodic Titania Nanotubes: A High Antipoison and Stable Catalyst System for Methanol Oxidation in Alkaline Media. J. Phys. Chem. C..

[18] Grimes C.A., Ong K.G., Varghese O.K., Yang X., Mor G., Paulose M., Dickey E.C., Ruan C., Pishko M.V., Kendig J.W. (2003). A Sentinel Sensor Network for Hydrogen Sensing. Sensors.

[19] Zhang Y., Fu W., Yang H., Qi Q., Zeng Y., Zhang T., Ge R., Zou G. (2008). Synthesis and Characterization of TiO_2_ Nanotubes for Humidity Sensing. Appl. Surf. Sci..

[20] Han C.H., Hong D.W., Kim I.J. (2007). Synthesis of Pd/Titanate Nanotube and Its Application to Catalytic Type Hydrogen Gas Sensor. Sens. Actuators B Chem..

[21] Joo S., Muto I., Hara N. (2010). Hydrogen Gas Sensor Using Pt- and Pd-Added Anodic TiO_2_ Nanotube Films. J. Electrochem. Soc..

[22] Steinhauer B., Kasireddy M.R., Radni J., Martin A. (2009). Development of Ni-Pd Bimetallic Catalysts for the Utilization of Carbon Dioxide and Methane by Dry Reforming. Appl. Catal. A Gen..

[23] Kumar M.K., Tan L.K., Gosvami N.N., Gao H. (2009). Conduction-Atomic Force Microscopy Study of H_2_ Sensing Mechanism in Pd Nanoparticles Decorated TiO_2_ Nanofilm. J. Appl. Phys..

[24] Liu X., Jaramillo T.F., Kolmakov A., Baeck S.H., Moskovits M., Stucky G.D., McFarland E.W. (2005). Synthesis of Au Nanoclusters Supported upon a TiO_2_ Nanotube Array. J. Mater. Res..

[25] Zhuo Y., Huang L., Ling L., Wang J. (2013). A Novel NO_2_ Sensor Based on TiO_2_ Nanotubes Array with *In-Situ* Au Decoration. J. Nanosci. Nanotechnol.

[26] Seo M.H., Yuasa M., Kida T., Huh J.S., Yamazoe N., Shimanoe K. (2011). Enhanced Gas Sensing Characteristics of Au-Loaded TiO_2_ Nanotube Sensors. Sens. Lett..

[27] Kolmakov A., Klenov D.O., Lilach Y., Stemmer S., Moskovits M. (2005). Enhanced Gas Sensing by Individual SnO_2_ Nanowires and Nanobelts Functionalized with Pd Catalyst Particles. Nano Lett..

[28] Henry C.R., Chapon C., Duriez C. (1991). Precursor State in the Chemisorption of CO on Supported Palladium Clusters. J. Chem. Phys..

[29] Tsu K., Boudart M. (1961). Recombination of Atoms at the Surface of Thermocouple Probes. Can. J. Chem..

[30] Boudart M. (1999). On the Nature of Spilt-Over Hydrogen. J. Mol. Catal. A Chem..

[31] Wang J., Lin Z. (2009). Anodic Formation of Ordered TiO_2_ Nanotube Arrays: Effects of Electrolyte Temperature and Anodization Potential. J. Phys. Chem. C..

[32] Regonini D., Jaroenworaluck A., Stevens R., Bowen C.R. (2010). Effect of Heat Treatment on the Properties and Structure of TiO_2_ Nanotubes: Phase Composition and Chemical Composition. Surf. Interf. Anal..

[33] Williams D.E. (1987). Solid State Gas Sensors.

[34] Varghese O.K., Gong D., Paulose M., Ong K.G., Grimes C.A. (2003). Hydrogen sensing using titania nanotubes. Sens. Actuators B Chem..

[35] Paulose M., Varghese O.K., Mor G.K., Grimes C.A., Ong K.G. (2006). Unprecedented ultra-high hydrogen gas sensitivity in undoped titania nanotubes. Nanotechnology.

[36] Yamazoe N., Shimanoe K. (2008). Theory of power laws for semiconductor gas sensors. Sens. Actuators B Chem..

[37] Yanga Z., Huang Y., Chena G., Guoc Z., Cheng S., Huang S. (2009). Ethanol Gas Sensor Based on Al-Doped ZnO Nanomaterial with Many Gas Diffusing Channels. Sens. Actuators B Chem..

[38] Vaishampayan M.V., Deshmukh R.G., Mulla I.S. (2008). Influence of Pd doping on morphology and PG response of SnO_2_. Sens. Actuators B Chem..

[39] Du A.J., Smith S.C., Yao X.D., Lu G.Q. (2007). Hydrogen Spillover Mechanism on a Pd-Doped Mg Surface as Revealed by ab initio Density Functional Calculation. J. Am. Chem. Soc..

[40] Jimenez-Cadena G., Riu J., Rius F.X. (2007). Gas sensors based on nanostructured materials. Analyst..

[41] Kapse V.D., Ghosh S.A., Raghumanshi F.C., Kapse S.D., Khandekar U.S. (2009). Nanocrystalline Ni_0.6_Zn_0.4_Fe_2_O_4_: A Novel Semiconducting Material for Ethanol Detection. Talanta.

[42] Kwon Y.J., Kim H.S., Lee S.M., Chin I.J., Seong T.Y., Lee W.I., Lee C. (2012). Enhanced ethanol sensing properties of TiO_2_ nanotube sensors. Sens. Actuators B Chem..

[43] Jin C.H., Park S.H., Kim H.S., Lee C. (2012). “Ultrasensitive multiple networked Ga_2_O_3_-core/ZnO-shell nanorod gas sensors”. Sens. Actuators B Chem..

