# Supplementary material for: Effects of Functionalization of TiO2 Nanotube Array Sensors with Pd Nanoparticles on Their Selectivity
Source: Sensors (Basel). 2014 Aug 27;14(9):15849–60. doi: 10.3390/s140915849 (PMC4208149; doi:10.3390/s140915849)
Supplement: Supplementary file 1 [file sensors-14-15849-s001.pdf]

OPEN ACCESS

sensors

ISSN 1424-8220
www.mdpi.com/journal/sensors

Supplementary Information

Effects of Functionalization of TiO_2 Nanotube Array Sensors with Pd Nanoparticles on Their Selectivity. *Sensors* 2014, *14*, 15849-15860

Sunghoon Park [1], Soohyun Kim [1], Suyoung Park [1], Wan In Lee [2] and Chongmu Lee [1,*]

[1] Department of Materials science and Engineering, Inha University, 253 Yonghyun-dong, Incheon 402-751, Korea; E-Mails: aju-ya@hanmail.net (S.H.P.); feel2779@naver.com (S.H.K.); dswimming@naver.com (S.Y.P.)

[2] Department of Chemistry, Inha University, 253 Yonghyun-dong, Incheon 402-751, Korea; E-Mail: wanin@inha.ac.kr

* Author to whom correspondence should be addressed; E-Mail: cmlee@inha.ac.kr; Tel.: +82-32-860-7536; Fax: +82-32-862-5546.

Table S1. Responses of the pristine TiO_2 and Pd-functionalized TiO_2 nanotube gas sensors to 10–3000 ppm C_2H_5OH gas at 200 °C and to 10–3000 ppm CH_3COCH_3 gas at 250 °C.

Concentration (ppm)	Response (%)			
	Ethanol		Acetone	
	Pd-TiO_2	TiO_2	Pd-TiO_2	TiO_2
10	297.42691	234.96621	212.5518	148.15856
50	795.87487	467.58524	384.5462	219.84535
100	1,589.99746	691.11812	862.1524	324.8482
200	3,132.46831	985.846	1,151.5184	472.84876
500	4,996.96311	1,333.62872	1,711.2546	661.84845
1,000	7,195.94023	1,656.53704	2,424.2546	825.15456
1,500	9,983.5159	2,009.15844	3,124.2435	1,184.5483
2,000	14,078.59089	2,354.96967	4,224.2454	1,342.56485
2,500	17,072.18865	2,693.10098	6,124.2546	1,488.4524
3,000	21,253.12128	2,877.39961	8,746.2454	1,635.84325

Table S2. Responses of the pristine TiO_2 and Pd-functionalized TiO_2 nanotube gas sensors to various gases.

Gas	Response (%)	
	Pd-TiO_2	TiO_2
CO (10 ppm)	1,871.65	1,722.71
H_2 (10,000 ppm)	2,018.87	1,539.26
NH_3 (100 ppm)	2,595.36	1,928.35
NO_2 (10 ppm)	6,122.4	3,184.27
Acetone (3,000 ppm)	8,746.25	16,358.4
Ethanol (3,000 ppm)	21,253.12	2,877.40

© 2014 by the authors; licensee Molecular Diversity Preservation International, Basel, Switzerland. This article is an open-access article distributed under the terms and conditions of the Creative Commons Attribution license (http://creativecommons.org/licenses/by/3.0/).
